# Supplementary material for: Impaired mitochondrial calcium efflux contributes to disease progression in models of Alzheimer’s disease
Source: Nat Commun. 2019 Aug 29;10:3885. doi: 10.1038/s41467-019-11813-6 (PMC6715724; doi:10.1038/s41467-019-11813-6)
Supplement: Supplementary file 1 — Supplementary Information [file 41467_2019_11813_MOESM1_ESM.pdf]

Supplementary Information

Impaired mitochondrial calcium efflux contributes to disease progression in models of Alzheimer's disease

Jadiya et al. Nature Communications. 2019.

Supplementary Methods

Membrane Rupture and cell viability assay. Equal numbers of N2a, APP*swe* and APP*swe* infected with Ad-NCLX for 48h were treated with Ionomycin, (1-5 μM) for 24h and an oxidizing agent *tert*-Butyl hydroperioxide (TBH) (10- 30 μM) for 14h and glutamate (NDMAR-agonist, neuroexcitotoxicity agent) (10- 50 μM) for 24 h. To measure number of viable cells, CellTiter-Blue Reagent (10μl/well in 96 well plate) is added directly to each well, incubated at 37°C for 2 hrs and the fluorescent signal at $(560(20)_{Ex}/590(10)_{Em})$.was measured using a Tecan Infinite M1000 Pro plate reader. Data is normalized to vehicle control to avoid any differences in cell numbers between the groups.

Citrate synthase activity. Citrate synthase activity was measured according to the method described in manual (BioVision Catalog #: K318). Briefly, mitochondria were isolated from brain cortex of mice, transferred to a 96-well plate (4μg/well) and brought to a volume of 50 μl with CS assay buffer provided in the kit. The reaction was initiated by adding of 50 μl of reaction mix (CS assay buffer, developer and substrate mix) to each well containing samples. Changes in absorbance at 412 nm in 25°C were measured in kinetic mode for 20 min. The results were presented as changes in activity within the groups (activity/μg protein/min).

Supplementary Table 1. Demographics of brain samples examined in this study.

Diagnosis	Gender	Age	Source
No dementia	Male	81	ASU Brain bank
No dementia	Male	78	ASU Brain bank
No dementia	Female	86	ASU Brain bank
No dementia	Female	82	ASU Brain bank
No dementia	Male	71	ASU Brain bank
No dementia	Male	80	ASU Brain bank
No dementia	Female	85	ASU Brain bank
Sporadic, non-familial AD	Female	86	ASU Brain bank
Sporadic, non-familial AD	Female	82	ASU Brain bank
Sporadic, non-familial AD	Female	80	ASU Brain bank
Sporadic, non-familial AD	Male	72	ASU Brain bank
Sporadic, non-familial AD	Male	75	ASU Brain bank
Sporadic, non-familial AD	Male	81	ASU Brain bank
Sporadic, non-familial AD	Female	77	ASU Brain bank

ASU: Arizona State University

Supplementary Table 2. List of primer sequences used in this study.

Primer sequences for Jadiya et al.		
**Gene** (mouse or human)	**Forward primer**	**Reverse primer**
Rps13	GCACCTTGAGAGGAACAGAA	GAGCACCCGCTTAGTCTTATAG
Mcu	GATGACGTGACGGTGGTTTA	GTCAGAGATAGGCTTGAGTGTG
Mcub	CGACAACATCGGCTTGACTA	GTGGAGCCACAGGATGAAATA
Micu1	AAGAACACTCCCTGCCATTT	GCCAGGGTCATCTGCATTAT
Micu2	GTGCTTTCTGGAGGGCTAAA	CTGCAAGTATTCCCTAAGCTATCA
Emre	GGGACACTCATCAGCAAGAA	CTCCCTGTGCCCTGTTAATC
NCLX (Slc8b1)	GCCATCTCCACTAACCTCAAA	GGGTCTGAGAAAGCCACTAAA
Hbb-bt genomic	GAAGCGATTCTAGGGAGCAG	GGAGCAGCGATTCTGAGTAGA
RPS13	CCTTCACAGATCGGTGTAATCC	TCAGGAGCAAGTCCCTTAGA
MCU	GGGGTACCCCACCAAAACAGCTATTC	CCCAAGCTTGGGTTCCGATCTGTCGG
MCUB	GTGTGAAGCTGTGTGGAAATG	CAAGGGAAGGCCATGTCTATAA
MICU1	AACATTCCTTGACTTCCCTCC	TCTGAATCTTGCTGTGTTCCC
MICU2	TGGAAGGATAAAACAATACATATGGG	GGAACATGAAGATGTCTGGAATTG
EMRE	CTGAGATGTGCTGTCCACTAAG	GAGACAACTGCACCAACTAGAA
NCLX (SLC8B1)	CAGAAAGGGAAGTGGAGAGTAAG	GCCATTAGCAGCACACAAAG
PGC-1α	CCGAGAATTCATGGAGCAAT	GTGTGAGGAGGGTCATCGTT
COXII	GCCGACTAAATCAAGCAACA	CAATGGGCATAAAGCTATGG

Supplementary Figures

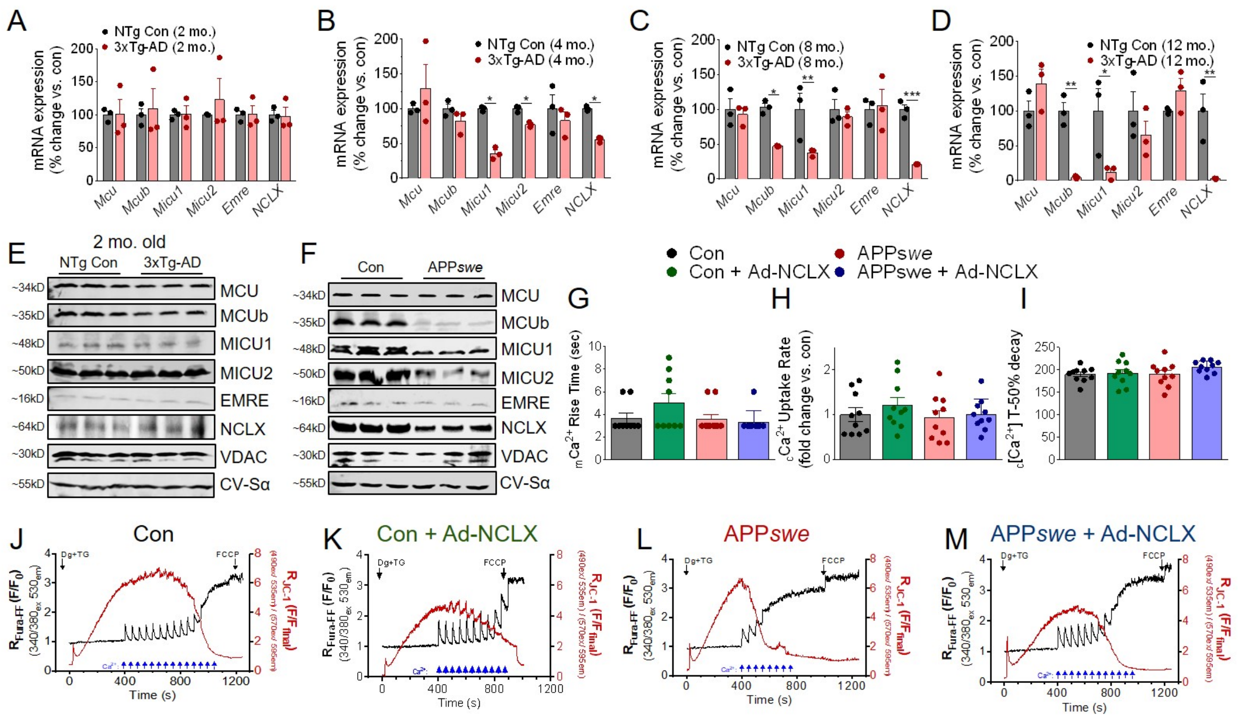

Supplementary Figure 1: $_{m}Ca^{2+}$ exchanger expression and $_{m}Ca^{2+}$ handling in AD.
(A) mRNA expression of $_{m}Ca^{2+}$ exchanger in brain tissue isolated from the frontal cortex of 2 mo. old 3xTg-AD mutant mice and age-matched outbred non-transgenic controls (NTg). n = 3 for both groups. **(B)** mRNA expression of $_{m}Ca^{2+}$ exchanger in brain tissue isolated from the frontal cortex of 4 month old 3xTg-AD mutant mice and age-matched outbred non-transgenic controls (NTg). n = 3 for both groups. **(C)** mRNA expression of $_{m}Ca^{2+}$ exchanger in brain tissue isolated from the frontal cortex of 8 month old 3xTg-AD mutant mice and age-matched outbred non-transgenic controls (NTg). n = 3 for both groups. **(D)** mRNA expression of $_{m}Ca^{2+}$ exchanger in brain tissue isolated from the frontal cortex of aged (12 mo.) 3xTg-AD mutant mice and outbred non-transgenic controls (NTg). n = 3 for both groups. **(E)** Western blots for $_{m}Ca^{2+}$ exchanger protein in 3xTg-AD mutant mice (2 mo.) and age-matched outbred non-transgenic controls (NTg). n = 3 for both groups. **(F)** Western blots for $_{m}Ca^{2+}$ exchanger proteins in neuroblastoma control cell line (N2a) vs. cells stably expressing cDNA encoding the APP Swedish mutant (K670N, M671L, APP*swe).* n = 3 for both groups. **(G)** Quantification of $_{m}Ca^{2+}$ rise time. **(H)** Fold change in $_{c}Ca^{2+}$ uptake rate of con + Ad-NCLX, APP*swe* and APP*swe* + Ad-NCLX vs. con (N2a) cells. n=10**. (I)** Time to 50% $_{c}Ca^{2+}$ transient decay (T-50%). n= 10 for all groups. **(J-M)** Representative traces for $_{m}Ca^{2+}$ retention capacity in con, con + Ad-NCLX, APP*swe* and APP*swe* cells infected with adenovirus encoding mitochondrial Na^{+}/Ca^{2+} exchanger (NCLX). Data was presented as mean ± SEM, ***p<0.001, **p<0.01, *p<0.05, one-way ANOVA, Sidak's multiple comparisons test.

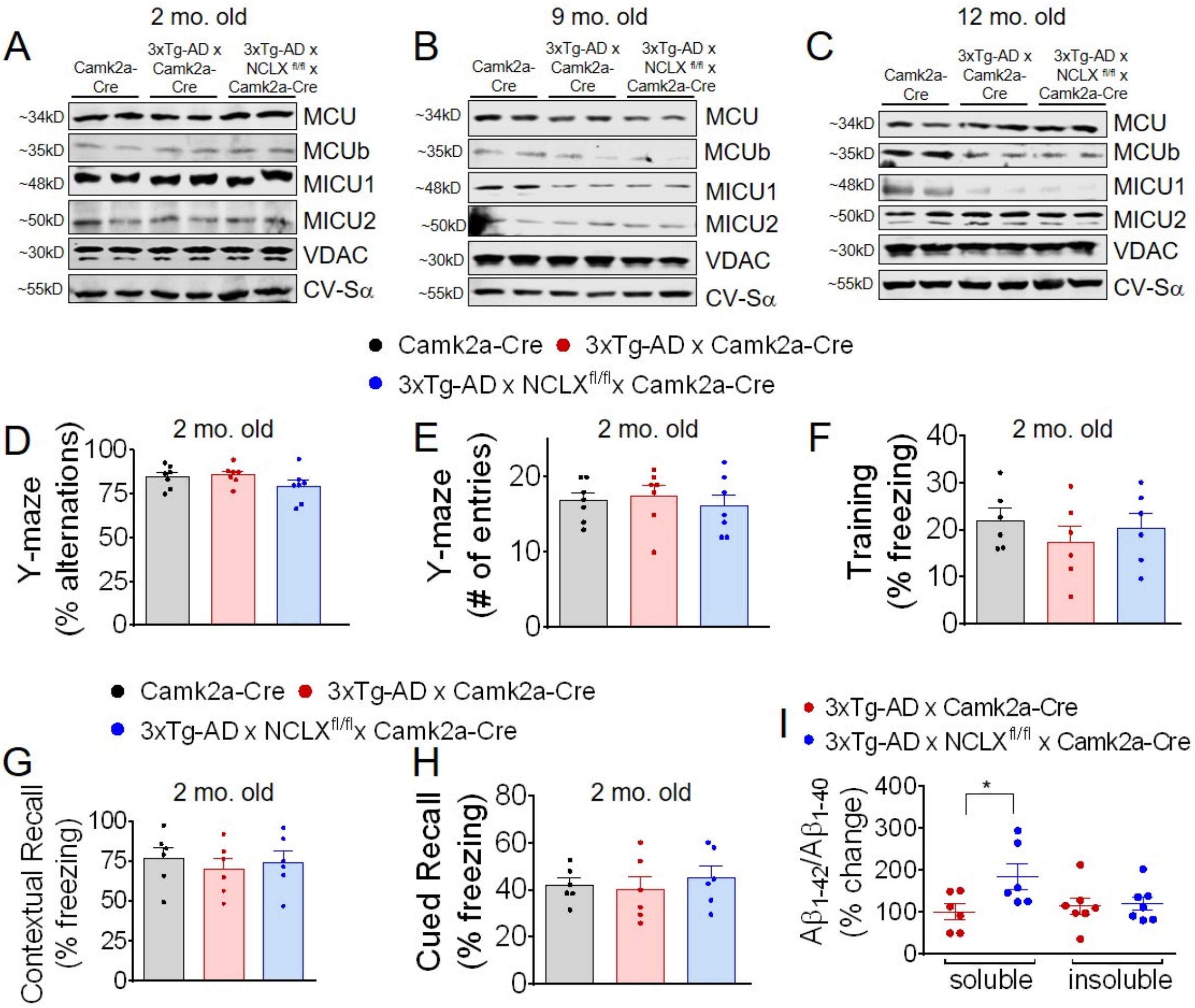

Supplementary Figure 2: NCLX deletion effect on the memory at the age of 2 mo.
(A-C) Western blots for NCLX expression and proteins associated with $_{m}Ca^{2+}$ exchange in tissue isolated from the hippocampus of 2, 9 and 12 mo. old 3xTg-AD x NCLX$^{fl/fl}$ x Camk2a-Cre mutant mice compared to age-matched control. MCU, mitochondrial calcium uniporter; MCUb, mitochondrial calcium uniporter β subunit; MICU1, mitochondrial calcium uptake 1; MICU2, mitochondrial calcium uptake 2; EMRE, essential MCU regulator; NCLX, Na^{+}/Ca^{2+} exchanger. Voltage dependent anion channel (VDAC) and oxidative phosphorylation component CV-Sα, complex V α subunit; were used as mitochondrial loading controls and has been shown in Fig. 2. **D-E)** Working memory was assessed in the Y-maze spontaneous alternation test in mice at the age of 2 mo. in Camk2a-Cre, 3xTg-AD x Camk2a-Cre and 3xTg-AD x NCLX$^{fl/fl}$ x Camk2a-Cre mice **(D)** Percentage spontaneous alternations. **(E)** Number of total arm entries. n = number of dots as shown for all groups. **(F-H)** Hippocampus and amygdala associated memory was assessed in the fear conditioning test in mice at the age of 2 mo. in Camk2a-Cre, 3xTg-AD x Camk2a-Cre and 3xTg-AD x NCLX$^{fl/fl}$ x Camk2a-Cre mice **(F)** Freezing responses in the training phase **(G)** Contextual recall freezing responses **(H)** Cued recall freezing responses. n= number of dots as shown for all groups. **(I)** Soluble (RIPA) and insoluble (formic acid extractable) $A\beta_{1-42}/A\beta_{1-40}$ ratio in brain cortex of 3xTg-AD x Camk2a-Cre and 3xTg-AD x NCLX$^{fl/fl}$ x Camk2a-Cre mice at the age of 12 mo. were measured by sandwich ELISA. Data was presented as mean ± SEM, ***p<0.001, **p<0.01, *p<0.05, one-way ANOVA, Sidak's multiple comparisons test.

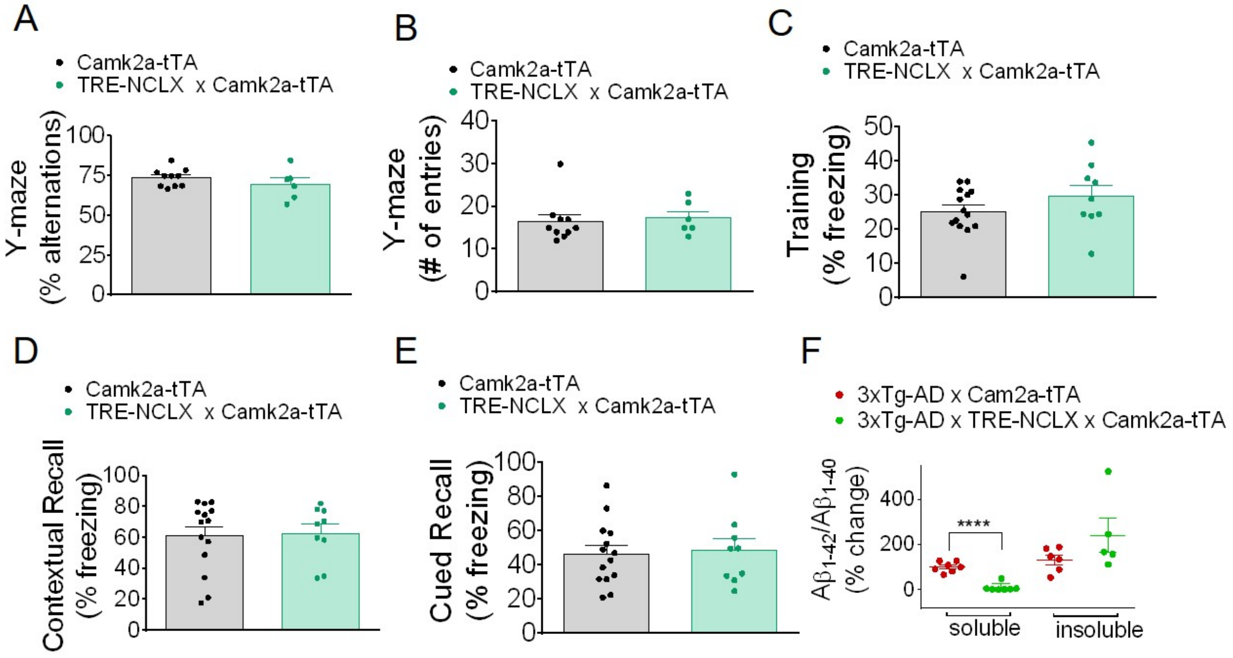

Supplementary Figure 3: NCLX overexpression effect on the memory in wild type mice **(A-B)** Working memory was assessed in the Y-maze spontaneous alternation test in mice at the age of 6 mo. in Camk2a-tTA and TRE-NCLX x Camk2a-tTA mice **(A)** Percentage spontaneous alternations. **(B)** Number of total arm entries. n = number of dots shown in Fig. **(C-E)** Hippocampus and amygdala associated memory was assessed in the fear conditioning test in mice at the age of 6 mo. in Camk2a-tTA and TRE-NCLX x Camk2a-tTA mice **(C)** Freezing responses in the training phase **(D)** Contextual recall freezing responses **(E)** Cued recall freezing responses. **(F)** Soluble (RIPA) and insoluble (formic acid extractable) $A\beta_{1-42}/A\beta_{1-40}$ ratio in brain cortex of 3xTg-AD x Camk2a-tTA and 3xTg-AD x TRE-NCLX x Camk2a-tTA mice at the age of 12 mo. were measured by sandwich ELISA. All data shown as mean ± SEM; one-way ANOVA, Sidak's multiple comparisons test, n= number of dots shown in Fig. for all groups. ***p<0.001, **p<0.01, *p<0.05.

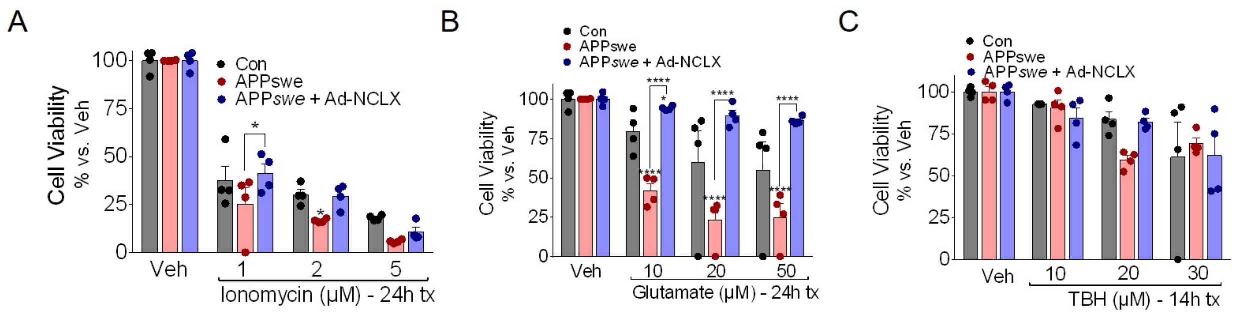

Supplementary Figure 4: Enhancing $_{m}Ca^{2+}$ efflux effect on cell viability
(A-C) N2a, APP*swe* and APP*swe* infected with Ad-NCLX for 48h were assessed for cell viability using Cell Titer Blue after treatment with **(A)** Ionomycin (Ca^{2+} overload, 1-5 μM), **(B)** glutamate (NDMAR-agonist, neuroexcitotoxicity agent, 10-50 μM). **(C)** *tert*-Butyl hydroperioxide (TBH, oxidizing agent, 10-30 μM), n = individual dots shown for each group. Data was presented as mean ± SEM, ***p<0.001, **p<0.01, *p<0.05, one-way ANOVA, Sidak's multiple comparisons test.

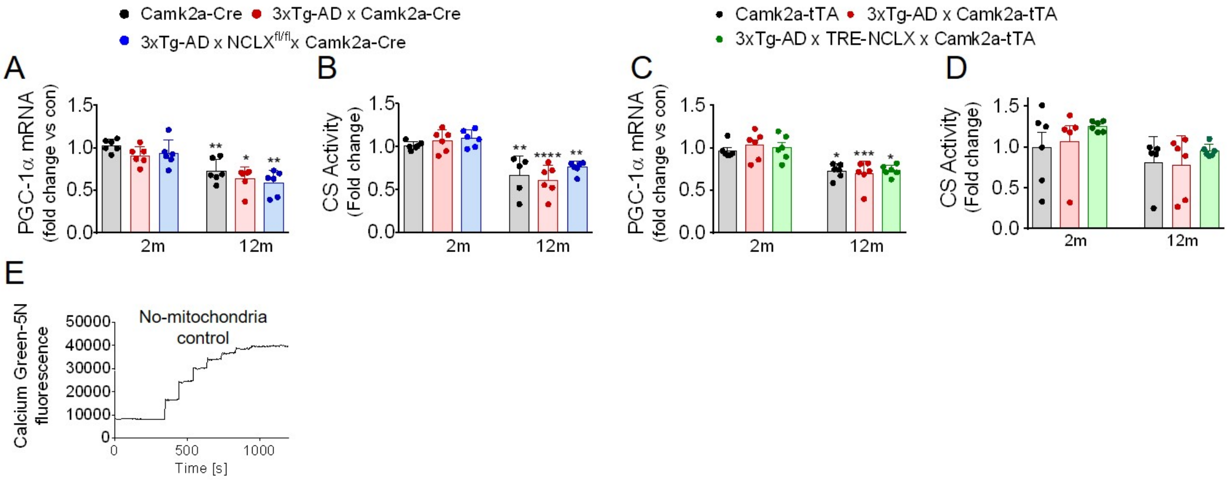

Supplementary Figure 5: Mitochondrial mass in deletion and overexpression of NCLX
(A) PGC-1α mRNA expression in tissue isolated from the brain cortex of 2 & 12 mo. old Camk2a-Cre, 3xTg-AD x Camk2a-Cre and 3xTg-AD x NCLX$^{fl/fl}$ x Camk2a-Cre mice expressed as fold-change vs. 2m old Camk2a-Cre con. n = 6 for all groups. **(B)** Citrate synthase activity in mitochondria isolated from the brain cortex of 2 & 12 mo. old Camk2a-Cre, 3xTg-AD x Camk2a-Cre and 3xTg-AD x NCLX$^{fl/fl}$ x Camk2a-Cre mice expressed as fold-change vs. 2m old Camk2a-Cre con. n = 6 for all groups. **(C)** PGC-1α mRNA expression in tissue isolated from the brain cortex of 2 & 12 mo. old Camk2a-tTA, 3xTg-AD x Camk2a-tTA and 3xTg-AD x TRE-NCLX x Camk2a-tTA mice expressed as fold-change vs. 2m old Camk2a-tTA con. n = 6 for all groups **(D)** Citrate synthase activity in mitochondria isolated from the brain cortex of 2 & 12 mo. old Camk2a-tTA, 3xTg-AD x Camk2a-tTA and 3xTg-AD x TRE-NCLX x Camk2a-tTA mice expressed as fold-change vs. 2m old Camk2a-Cre con. n = 6 for all groups. **(E)** A standard curve of Ca^{2+} (5-50μm) in experimental intracellular buffer to quantify actual Ca^{2+} content as shown in Fig. 5A-D. Data was presented as mean ± SEM, ***p<0.001, **p<0.01, *p<0.05, one-way ANOVA, Sidak's multiple comparisons test.

Full length blots for Figure 1A

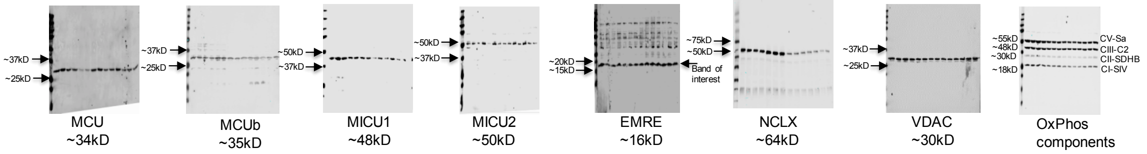

Full length blots for Figure 1C

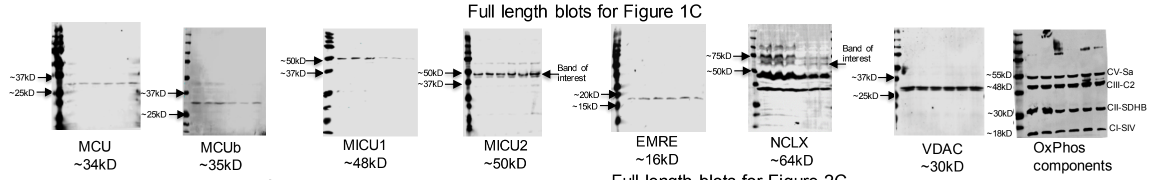

Full length blots for Figure 1E

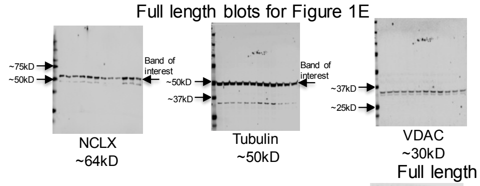

Full length blots for Figure 2C

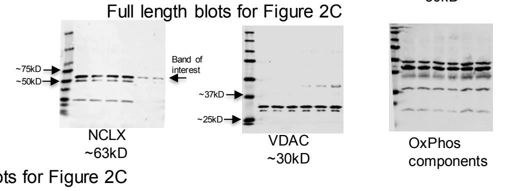

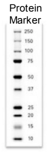

Full length blots for Figure 2C

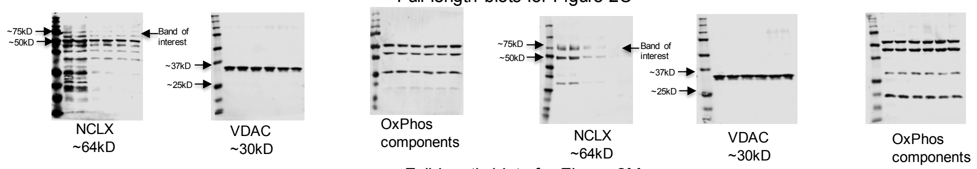

Full length blots for Figure 2M

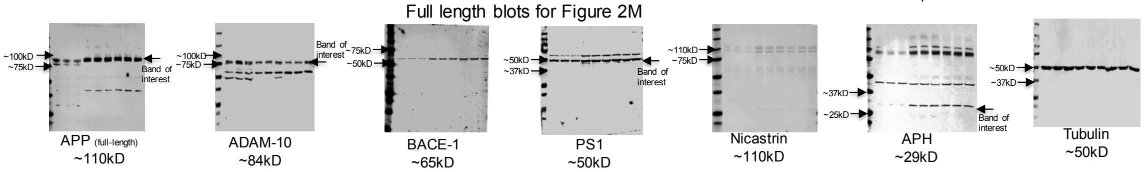

Full length blots for Figure 2N

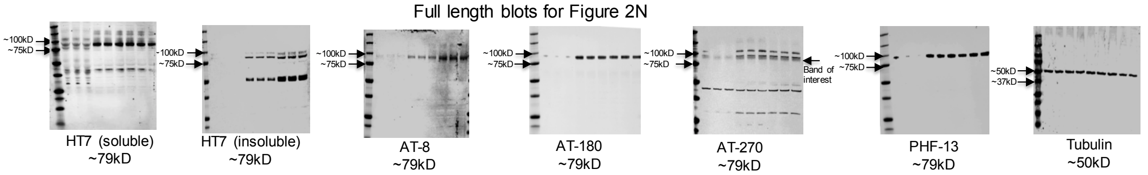

Full length blots for Figure 3C

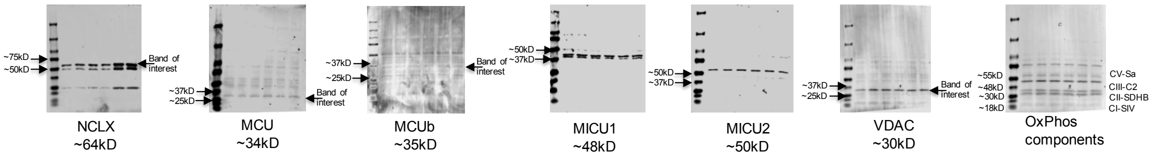

Full length blots for Figure 3M

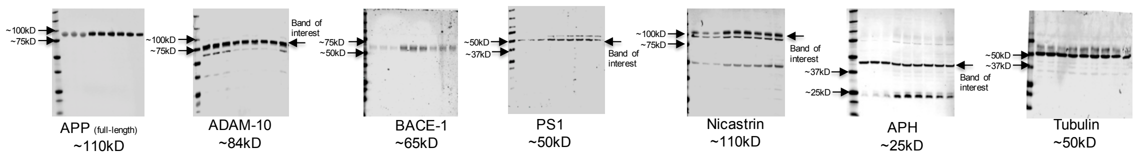

Full length blots for Figure 3N

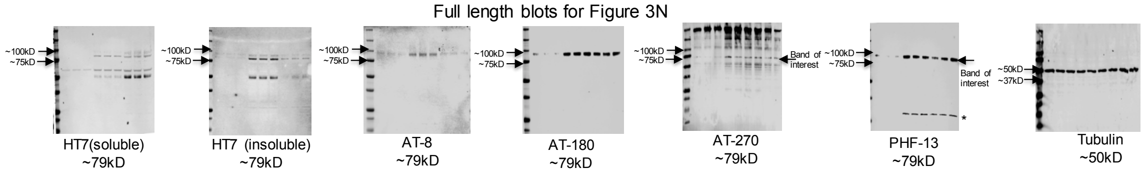

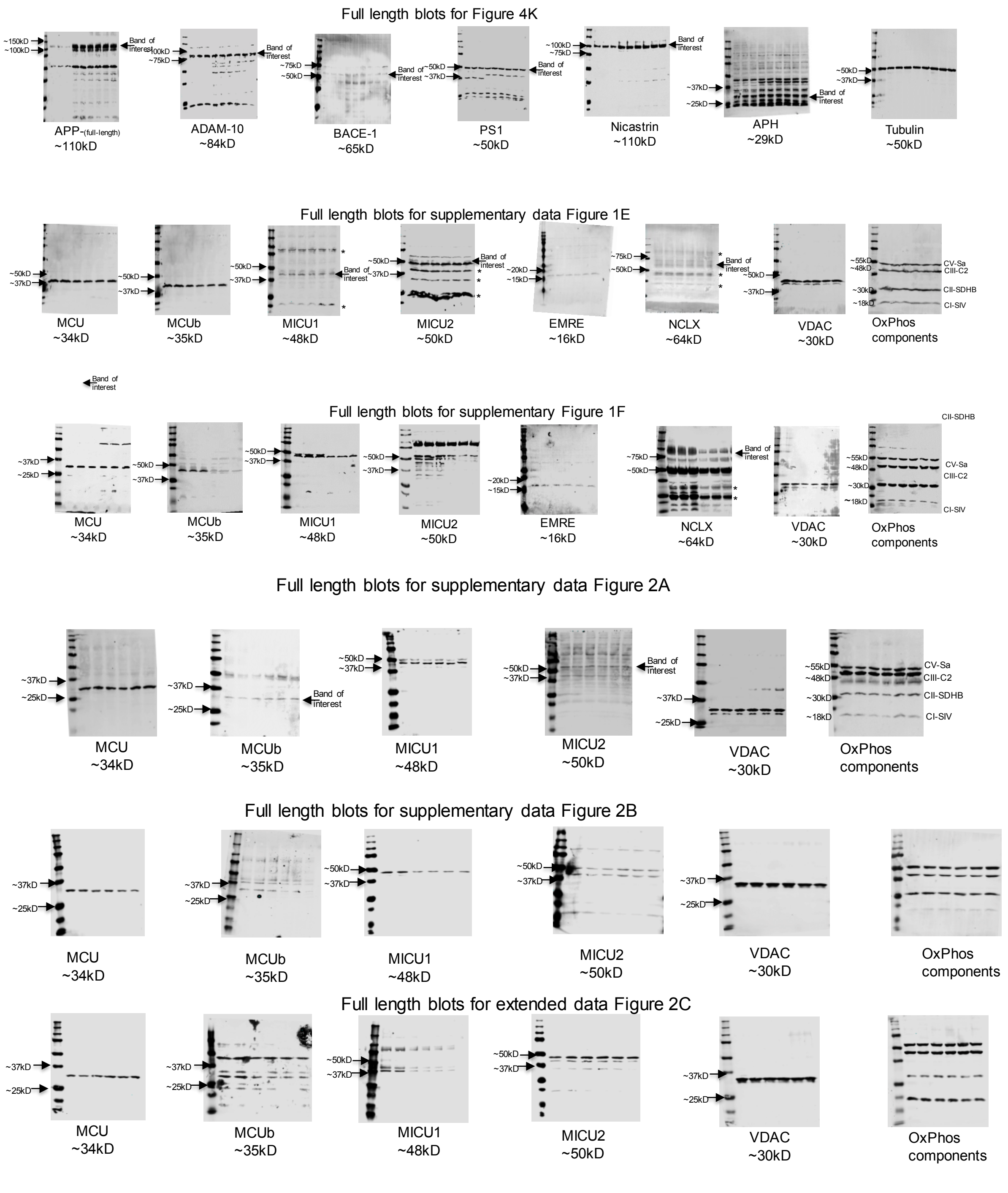

Supplementary Figure 6: Full-length Western blots.

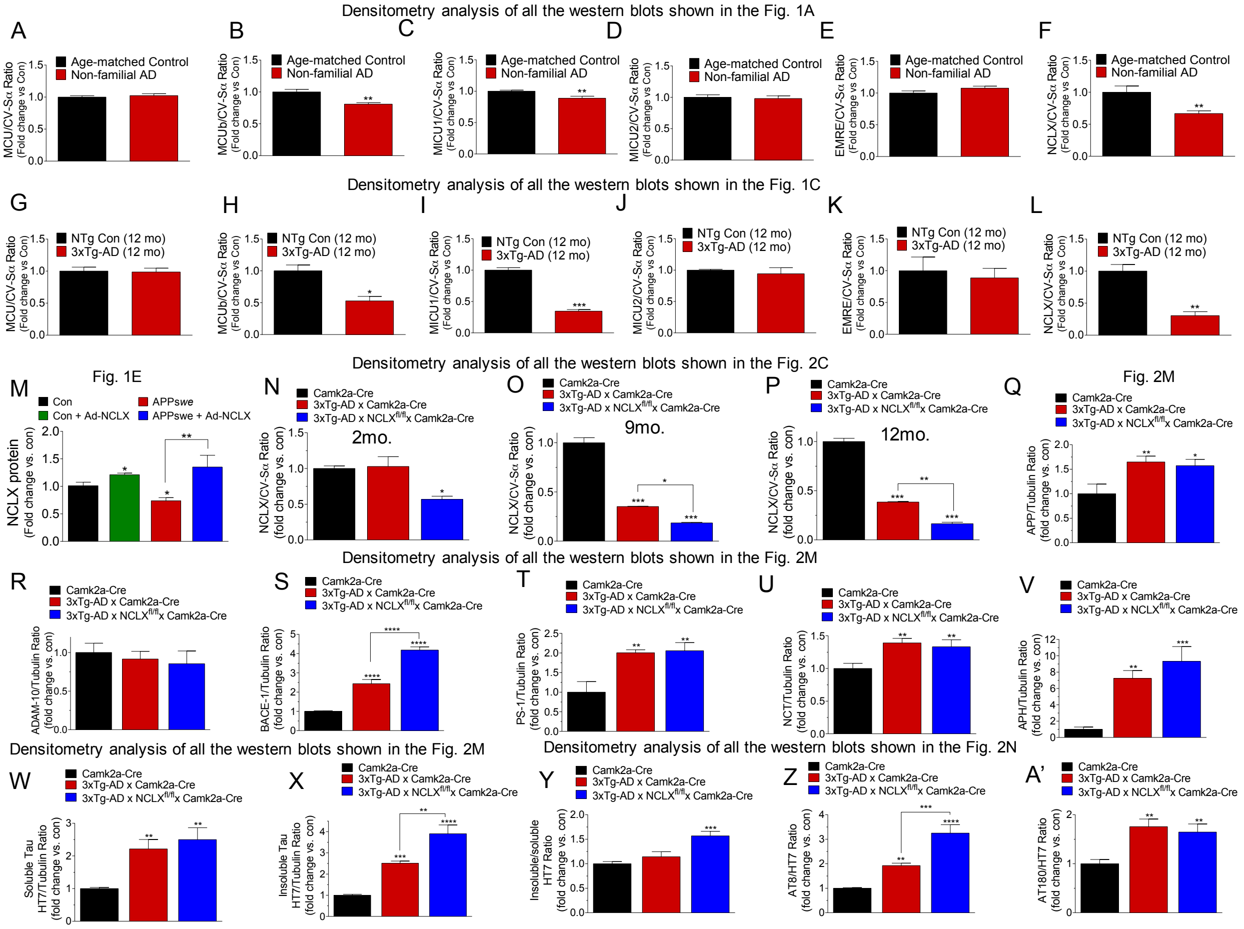

Densitometry analysis of all the western blots shown in the Fig. 1A
A
B
C
D
E
F
Age-matched Control
Non-familial AD
MCU/CV-Sα Ratio (Fold change vs Con)
MCUb/CV-Sα Ratio (Fold change vs Con)
MICU1/CV-Sα Ratio (Fold change vs Con)
MICU2/CV-Sα Ratio (Fold change vs Con)
EMRE/CV-Sα Ratio (Fold change vs Con)
NCLX/CV-Sα Ratio (Fold change vs Con)
Densitometry analysis of all the western blots shown in the Fig. 1C
G
H
I
J
K
L
NTg Con (12 mo)
3xTg-AD (12 mo)
Densitometry analysis of all the western blots shown in the Fig. 2C
M
Fig. 1E
Con
Con + Ad-NCLX
APPswe
APPswe + Ad-NCLX
NCLX protein (Fold change vs. con)
N
O
P
Q
Fig. 2M
Camk2a-Cre
3xTg-AD x Camk2a-Cre
3xTg-AD x NCLX fl/fl x Camk2a-Cre
2mo.
9mo.
12mo.
NCLX/CV-Sα Ratio (fold change vs. con)
APP/Tubulin Ratio (fold change vs. con)
Densitometry analysis of all the western blots shown in the Fig. 2M
R
S
T
U
V
ADAM-10/Tubulin Ratio (fold change vs. con)
BACE-1/Tubulin Ratio (fold change vs. con)
PS-1/Tubulin Ratio (fold change vs. con)
NCT/Tubulin Ratio (fold change vs. con)
APH/Tubulin Ratio (fold change vs. con)
Densitometry analysis of all the western blots shown in the Fig. 2M
Densitometry analysis of all the western blots shown in the Fig. 2N
W
X
Y
Z
A'
Soluble Tau HT7/Tubulin Ratio (fold change vs. con)
Insoluble Tau HT7/Tubulin Ratio (fold change vs. con)
Insoluble/soluble HT7 Ratio (fold change vs. con)
AT8/HT7 Ratio (fold change vs. con)
AT180/HT7 Ratio (fold change vs. con)

Densitometry analysis of all the western blots shown in the Fig. 2N

Densitometry analysis of all the western blots shown in the Fig. 3C

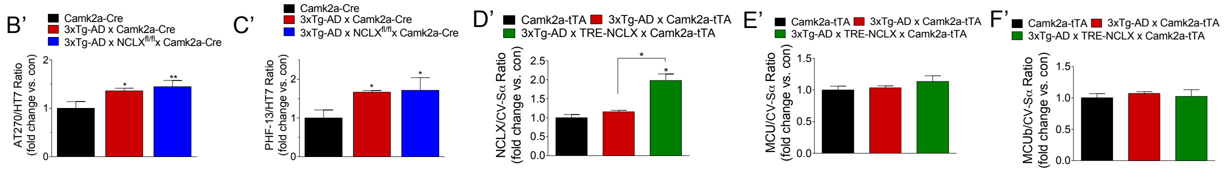

Densitometry analysis of all the western blots shown in the Fig. 3M

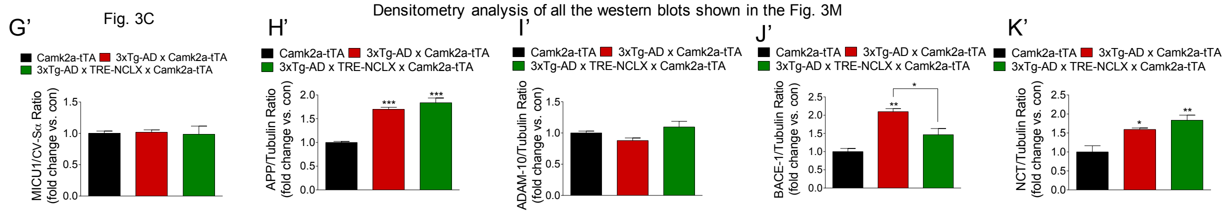

Densitometry analysis of all the western blots shown in the Fig. 3N

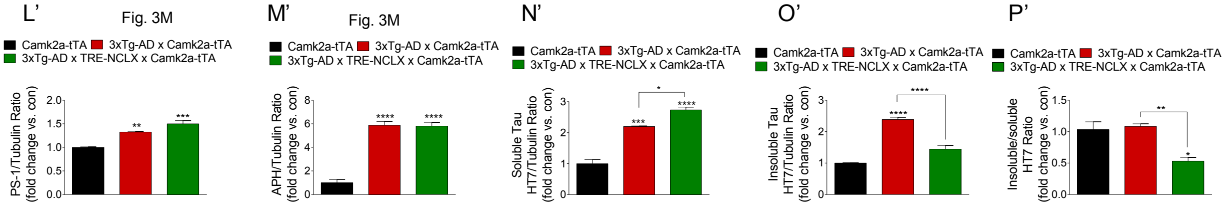

Densitometry analysis of all the western blots shown in the Fig. 3N

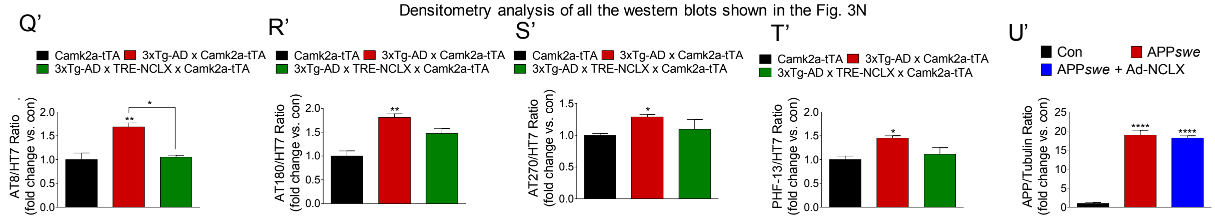

Densitometry analysis of all the western blots shown in the Fig. 4K

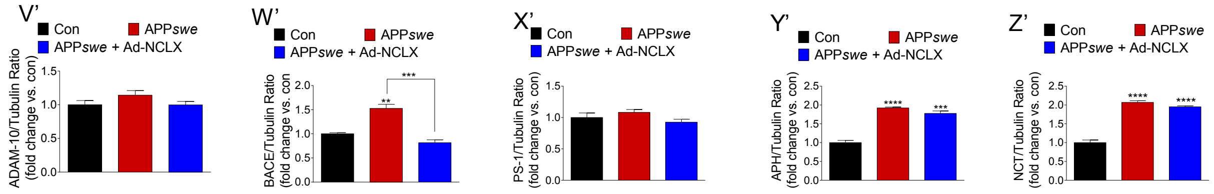

Densitometry analysis of all the western blots shown in the supplementary Fig. 1E

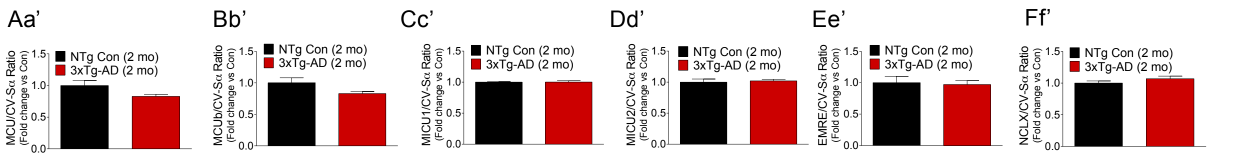

Densitometry analysis of all the western blots shown in the supplementary Fig. 1F

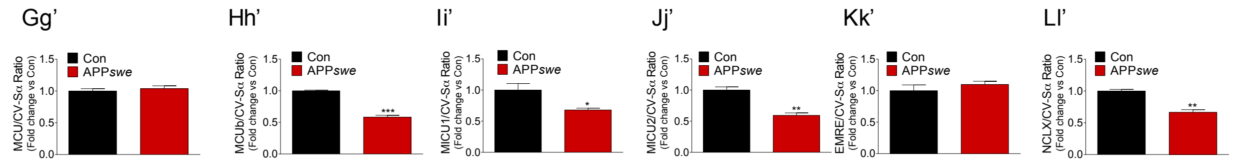

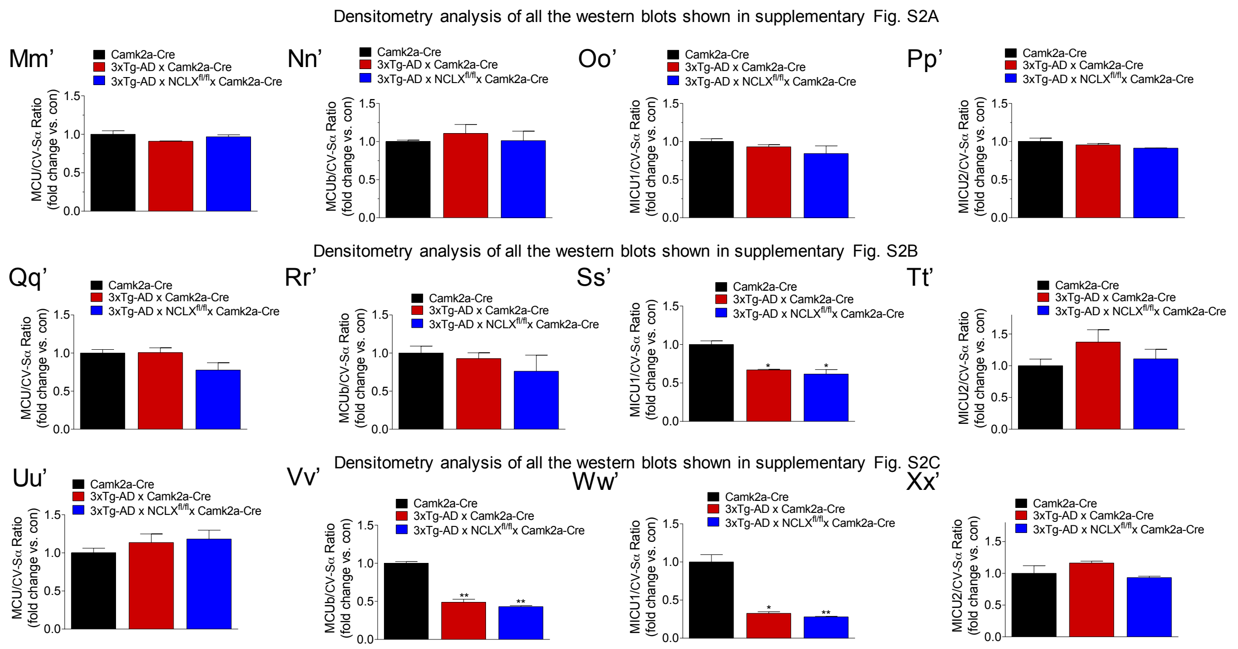

Supplementary Figure 7: Densitometry analysis of Western blots.
