# Supplementary material for: Impaired mitochondrial calcium efflux contributes to disease progression in models of Alzheimer’s disease
Source: Nat Commun. 2019 Aug 29;10:3885. doi: 10.1038/s41467-019-11813-6 (PMC6715724; doi:10.1038/s41467-019-11813-6)
Supplement: Supplementary file 3 — Reporting Summary [file 41467_2019_11813_MOESM3_ESM.pdf]

nature research

Corresponding author(s): John W Elrod

Last updated by author(s): Jul 8, 2019

Reporting Summary

Nature Research wishes to improve the reproducibility of the work that we publish. This form provides structure for consistency and transparency in reporting. For further information on Nature Research policies, see Authors & Referees and the Editorial Policy Checklist.

Statistics

For all statistical analyses, confirm that the following items are present in the figure legend, table legend, main text, or Methods section.

n/a	Confirmed	
☐	☒	The exact sample size (*n*) for each experimental group/condition, given as a discrete number and unit of measurement
☐	☒	A statement on whether measurements were taken from distinct samples or whether the same sample was measured repeatedly
☐	☒	The statistical test(s) used AND whether they are one- or two-sided<br>*Only common tests should be described solely by name; describe more complex techniques in the Methods section.*
☐	☒	A description of all covariates tested
☐	☒	A description of any assumptions or corrections, such as tests of normality and adjustment for multiple comparisons
☐	☒	A full description of the statistical parameters including central tendency (e.g. means) or other basic estimates (e.g. regression coefficient) AND variation (e.g. standard deviation) or associated estimates of uncertainty (e.g. confidence intervals)
☐	☒	For null hypothesis testing, the test statistic (e.g. *F*, *t*, *r*) with confidence intervals, effect sizes, degrees of freedom and *P* value noted<br>*Give P values as exact values whenever suitable.*
☒	☐	For Bayesian analysis, information on the choice of priors and Markov chain Monte Carlo settings
☒	☐	For hierarchical and complex designs, identification of the appropriate level for tests and full reporting of outcomes
☒	☐	Estimates of effect sizes (e.g. Cohen's *d*, Pearson's *r*), indicating how they were calculated

Our web collection on statistics for biologists contains articles on many of the points above.

Software and code

Policy information about availability of computer code

Data collection	For fear conditioning a foot shock and freezing behavior was recorded using the Freeze® Software system and Start Fear Systems; Harvard Apparatus, Holliston, MA, USA.
Data analysis	As given in the manuscript.

For manuscripts utilizing custom algorithms or software that are central to the research but not yet described in published literature, software must be made available to editors/reviewers. We strongly encourage code deposition in a community repository (e.g. GitHub). See the Nature Research guidelines for submitting code & software for further information.

Data

Policy information about availability of data

All manuscripts must include a data availability statement. This statement should provide the following information, where applicable:

- Accession codes, unique identifiers, or web links for publicly available datasets
- A list of figures that have associated raw data
- A description of any restrictions on data availability

As given in the manuscript and supplementary information files.

Field-specific reporting

Please select the one below that is the best fit for your research. If you are not sure, read the appropriate sections before making your selection.

☒ Life sciences ☐ Behavioural & social sciences ☐ Ecological, evolutionary & environmental sciences

Life sciences study design

All studies must disclose on these points even when the disclosure is negative.

Sample size	No statistical method is used to predetermine the sample size. However, our sample sizes are similar to those reported earlier by us and by others.
Data exclusions	No samples or animals were excluded from the study.
Replication	Experiments were replicated multiple times with reproducible results indicated in the figure legends.
Randomization	Animals were randomly assigned to each experimental groups.
Blinding	Experiments were performed in blinded fashion whenever possible. For example, mice were ear tagged to mask genotype. Data analysis were performed blindly.

Reporting for specific materials, systems and methods

We require information from authors about some types of materials, experimental systems and methods used in many studies. Here, indicate whether each material, system or method listed is relevant to your study. If you are not sure if a list item applies to your research, read the appropriate section before selecting a response.

Materials & experimental systems

n/a	Involved in the study	
☐	☒	Antibodies
☐	☒	Eukaryotic cell lines
☒	☐	Palaeontology
☐	☒	Animals and other organisms
☐	☒	Human research participants
☒	☐	Clinical data

Methods

n/a	Involved in the study	
☒	☐	ChIP-seq
☒	☐	Flow cytometry
☒	☐	MRI-based neuroimaging

Antibodies

Antibodies used	Detailed information is provided in the methods section of the manuscript.
Validation	As given in the manuscript.

Eukaryotic cell lines

Policy information about cell lines

Cell line source(s)	As given in the manuscript.
Authentication	Cell lines were authenticated by Western blotting and previously reported by other groups in detail. None of the cell lines are original to this work.
Mycoplasma contamination	Cell lines originally tested negative for the mycoplasma contamination.
Commonly misidentified lines (See ICLAC register)	None

Animals and other organisms

Policy information about studies involving animals; ARRIVE guidelines recommended for reporting animal research

Laboratory animals	As detailed in the manuscript.
Wild animals	N/A
Field-collected samples	N/A
Ethics oversight	Institutional Animal Care & Use Committee (IACUC) of Temple University

Note that full information on the approval of the study protocol must also be provided in the manuscript.

Human research participants

Policy information about studies involving human research participants

Population characteristics	As given in the manuscript.
Recruitment	As given in the manuscript.
Ethics oversight	As given in the manuscript.

Note that full information on the approval of the study protocol must also be provided in the manuscript.
